# Identification of potential neuroprotective compound from *Ganoderma lucidum* extract targeting microtubule affinity regulation kinase 4 involved in Alzheimer's disease through molecular dynamics simulation and MMGBSA

**DOI:** 10.1002/agm2.12232

**Published:** 2022-12-12

**Authors:** Faizan Ahmad, Gagandeep Singh, Hemant Soni, Smriti Tandon

**Affiliations:** ^1^ Department of Medical Elementology and Toxicology Jamia Hamdard University Delhi India; ^2^ Section of Microbiology, Central Ayurveda Research Institute, Jhansi CCRAS, Ministry of AYUSH Delhi India; ^3^ Kusuma School of Biological Sciences Indian Institute of Technology Delhi India

**Keywords:** Alzheimer's disease, *Ganoderma lucidum*, MD simulation, MMGBSA

## Abstract

**Objective:**

Alzheimer's disease (AD) is one of the most prevalent neurological ailments, affecting around 50 million individuals globally. The condition is characterized by nerve cell damage due to the formation of amyloid‐beta plaques and neurofibrillary tangles. Only a few US Food and Drug Administration (FDA)‐approved medications are available in the market which are devoid of side effects, thus, making it imperative to investigate new alternatives for countering this disease. According to a recent study, microtubule affinity regulation kinase 4 (MARK4) is attributed as one of the most promising drug targets for AD, thus, being selected for this study. Compounds from *Ganoderma lucidum* (Reishi mushroom) extracts were selected to be used as ligands for this study.

**Methods:**

In this study, the five most potent compounds from *Ganoderma lucidum* were selected and their absorption, distribution, metabolism, excretion, and toxicity (ADMET) analysis was performed, followed by molecular docking, and molecular dynamics simulation of each compound with MARK4 and supported by molecular mechanics generalized born surface area (MMGBSA) binding free energy calculations.

**Results:**

The promising compounds were selected based on their ADMET profile and interactions with the active site residues of MARK4. Based on docking scores of −9.1 and −10.3 kcal/ mol, respectively, stability assessment by molecular dynamics simulation, and MMGBSA calculations, ganoderic acid A and ganoderenic acid B were found to be the most promising compounds against MARK4 which will require further in vitro and in vivo validations.

**Conclusion:**

Through this study, it is suggested that ganoderic acid A and ganoderenic acid B might be a class of promising compounds against AD, based on computational research, and can be further studied for preclinical and clinical studies.

## Graphical abstract

Alzheimer's disease is the world's most common neurological disorder. Microtubule affinity regulation kinase 4 (MARK4) is currently one of the most promising drug targets for Alzheimer's disease. *Ganoderma lucidum*, which is also known as “Reishi mushroom,” from which five potential compounds were selected. Absorption, distribution, metabolism, excretion, and toxicity (ADMET) analysis followed by molecular docking and molecular dynamics for each complex was performed. Based on molecular dynamics simulation and molecular mechanics generalized born surface area (MMGBSA) results, this study revealed that ganoderic acid A and ganoderenic acid B are the most promising compounds against Alzheimer's disease.

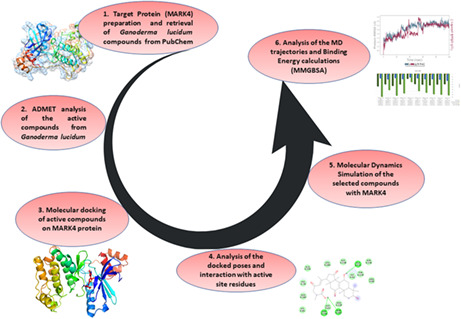

## 1 INTRODUCTION

The Alzheimer's disease (AD) is one of the most common neurodegenerative disorders, with symptoms including memory loss, mood swings, and functional difficulties.^1^ Dementia, which includes AD, affects 50 million individuals globally, most of whom are senile. According to the World Health Organization (WHO), AD is the world's fifth leading cause of death, with the number of fatalities anticipated to triple by 2050 if current trends continue.^2^ AD is diagnosed using brain imaging, physical and cognitive evaluations, laboratory tests, and medical history.^3^ The risk factors for AD include apolipoprotein E4 (APOE4) genotype and traumatic brain injury, family history and age, obesity, diabetes, hypercholesterolemia, hypertension, and a lack of education.^4^ Therefore, pharmaceutical and nonpharmacological treatments, such as physical, social, and cognitive activities, should be incorporated into the therapy regimen. Sodium valproate and lithium are the medications used to treat mild to moderate cognitive dysfunction.^5^, ^6^ Nonsteroidal anti‐inflammatory drugs (NSAIDs), such as naproxen and ibuprofen, reduce neuroinflammation and prevent neurodegeneration.^7^, ^8^ Some of the drugs that have failed clinical trials include Ganstigmine, Metrifonate, Lecithin, Ibuprofen, Rofecoxib, and Latrepirdine. Headaches, nausea, vomiting, neuromuscular dysfunction, and breathing issues have all been reported as side effects of these medications.^5^, ^9^ Only five US Food and Drug Administration (FDA)‐approved medicines are currently available to treat AD symptoms. Aducanumab was recently approved in the year 2021. Memantine (N‐methyl‐D‐aspartate receptor [NMDAR] inhibitor), was approved 10 years ago. Rivastigmine, donepezil (both cholinesterase inhibitors [ChEls]), and galantamine (an NMDAR antagonist) are the remaining drugs.^3^, ^10^

In this study, microtubule affinity regulation kinase 4 (MARK4), a member of the serine/threonine kinase family, primarily involved in microtubule assembly and the cell cycle, was selected as a target protein. The crystal structure of MARK4 was first observed in a complex with the pyrazolopyrimidine inhibitor. MARK4 overexpression has been observed in the initiation of neurodegenerative diseases, such as AD and Parkinson's disease (PD). Overexpressed MARK4 is responsible for the phosphorylation of Tau protein at the Ser262 site, which is required for the binding of microtubules to tau proteins. Co‐localization of phosphorylated MARK4 with phospho‐tau Ser262 in GVDs accumulating in AD samples has been reported. Studies have shown an association of MARK4 expression with neurodegenerative diseases, such as AD and PD, with MARK4 being present in significantly higher expression levels in neurodegenerative disease cases. These results hypothesized a potent MARK4 inhibitor can be designed to be used as an inhibitor against neurodegenerative diseases.^11^ In this computational study, *Ganoderma lucidum*, also known as “Reishi mushroom,” which comes under the category of medicinal mushroom, was selected. *Ganoderma lucidum* has been known to possess immune‐stimulating activities, anti‐inflammatory, and anti‐allergenic properties. This study aims to find a promising compound from *Ganoderma lucidum*, which can be used for preclinical and clinical trials against neurodegenerative disorders. In this study, the five most promising molecules from the Reishi mushroom: ganoderic acid A, ganoderic acid D, ganoderic acid F, ganoderenic acid B, and ganoderenic acid D^12^ were selected. Molecular docking for the five compounds mentioned above was performed, followed by molecular dynamics simulation for all five compounds. Out of these five, two compounds, namely, ganoderic acid A and ganoderenic acid B, were found to be most potent inhibitors against the MARK4 protein.

## 2 METHODS

### 2.1 ADMET (absorption, distribution, metabolism, excretion and toxicity analysis

SwissADME webserver was used to estimate the pharmacokinetic and pharmacological properties of the five compounds from *Ganoderma lucidum*.

### 2.2 Molecular docking

Molecular docking was performed with AutoDock 4.2.6, using the standard procedures. The 3D coordinates of the MARK4 protein were retrieved from the RCSB database with the PDB ID 5ES1 and five compounds of *Ganoderma lucidum* from the PubChem database. A grid box with x, y, and z directions was generated, keeping the grid spacing at 0.375 Å, and flexible multiple ligand docking was performed using a Perl script. The outputs were analyzed and visualized in Discovery Studio Visualizer.

### 2.3 Molecular dynamics

Desmond, a program by Schrodinger LLC,^13^ was used to model molecular dynamics in 100 ns. Structural studies were performed on the protein‐ligand complex as the first step in the molecular dynamics model preparation. Under static conditions, molecular connectivity studies can predict the binding state of ligands. The substrate attachment creates a static picture of the crucial position of an individual molecule in the active site of the protein.^14^ At the same time, the molecular dynamics simulation uses Newton's classical equation of motion to calculate the movement of atoms over time. The binding state of the ligands in the physiological environment was predicted using simulations.^15^, ^16^ Pretreatment of the accompanying protein‐ligand complex using the Protein Preparation Wizard or Maestro software to optimize and minimize the structure of the protein‐ligand complex was conducted. All systems were prepared using the System Builder tool. Transferable Intermolecular Interaction Potential 3 Points (TIP3P) was chosen as the orthorhombic solvent model because of its simplicity. Simulations were performed using the OPLS 2005 force field.^17^ 0.15 M sodium chloride (NaCl) was applied to simulate physiological conditions. The NPT ensemble was used throughout the simulation, with a temperature of 300 K and a pressure of 1 atm.

### 2.4 Molecular mechanics generalized born and surface area calculations

The binding free energies (∆G_bind_) of the anchored complexes were calculated using the Molecular Mechanics Generalized Born Surface Area (MMGBSA) module (Schrodinger suite, LLC). Binding free energies were calculated using OPLS 2005 force field, VSGB solvent model, and rotamer search methods.^18^ After performing Molecular Dynamics simulations (MDS), a period of 10 ns was used to select the MD orbital frame. The total free binding energy is calculated using the below‐mentioned formula:

$${\text{∆G}}_{\text{bind}}={\text{∆G}}_{\text{complex}}–\left({\text{∆G}}_{\text{protein}}+{\text{∆G}}_{\text{ligand}}\right).$$

where ∆G_bind_, binding free energy; ∆G_complex_, free energy of the complex; ∆G_protein_, free energy of the target protein, and ∆G_ligand_, free energy of the ligand.

## 3 RESULTS

### 3.1 ADME absorption, distribution, metabolism, a excretion analysis

The absorption, distribution, metabolism, excretion, and toxicity (ADMET) analysis of the selected molecules was done. It describes the absorption, distribution, drug metabolism, and excretion of a substance or medication.^19^, ^20^ Solubility and permeability are critical characteristics that influence the oral absorption rate.^21^, ^22^ As soon as the medication is dissolved, it circulates throughout the body and is distributed throughout different systems. Drug distribution into tissues and organs is governed by pharmacokinetic characteristics, such as plasma protein binding, lipophilicity, and phospholipids, three essential variables to consider.^23^, ^24^ Drug metabolism is the process by which a drug molecule is broken down or altered enzymatically, followed by excretion.^25^ The most important metric for determining pharmaceutical excretion in humans is renal clearance, which is essential for assessing pharmaceutical excretion in humans.^26^ Lipinski's rule of five, also referred to as the Pfizer rule of five, requires that oral pharmaceuticals have a molecular weight of less than 500, no more than five hydrogen bond donors, and 10 hydrogen bond acceptors, and a distribution ratio of less than five to be evaluated for approval.^26^, ^27^, ^28^ All the five compounds follow Lipinski's rule of five except the molecular weight. The result of the ADMET analysis of the five compounds is mentioned in Table 1.

**TABLE 1:** ADMET analysis result of five compounds of *Ganoderma lucidum* extract

Parameters	Ganoderic acid A	Ganoderic acid D	Ganoderic acid F	Ganoderenic acid B	Ganoderenic acid D
Molecular weight	516.67 g/mol	514.65 g/mol	570.67 g/mol	514.65 g/mol	512.63 g/mol
Number of H‐bond acceptor	7	7	9	7	7
Number of H‐bond donor	3	2	1	3	2
CYP1A2 inhibitor	No	No	No	No	Yes
CYP2C19 inhibitor	No	No	No	No	No
CYP2C9 inhibitor	No	No	No	No	No
CYP2D6 inhibitor	No	No	No	No	No
CYP3A4 inhibitor	Yes	No	No	Yes	No
Log Kp (skin permeation)	−7.90 cm/s	−8.10 cm/s	−8.16 cm/s	−7.76 cm/s	−7.98 cm/s
Lipinski's rule of five	Yes	Yes	Yes	Yes	Yes

Abbreviation: ADMET, absorption, distribution, metabolism, excretion, and toxicity.

### 3.2 Molecular docking

**FIGURE 1:**
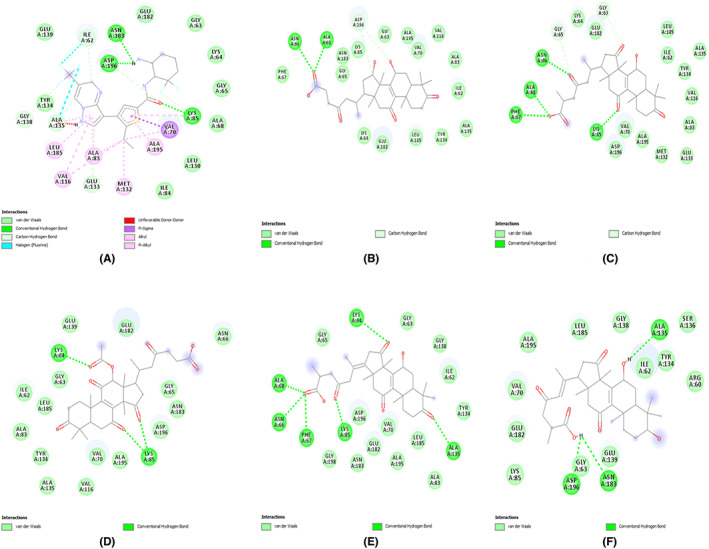
The 2D interaction diagram of the docked ligands with the active site residues of the target protein MARK4 (A) Co‐crystal ligand 5RC, (B) 471002, (C) 14109405, (D) 23247895, (E) 76378890, (F) 78074039.

The selected five molecules from the *Ganoderma lucidum* were docked at the active site of the protein MARK4. The active site of MARK4 consists of the residues Ile62, Lys64, Val70, Ala83, Lys85, Glu133, Tyr134, Ala135, Gly138, Glu182, Asn183, and Asp196, as analyzed by using the crystal structure of MARK4 (5ES1) with the co‐crystallized ligand 5RC. The binding affinity of the five compounds lies between −8.3 and −10.3 kcal/mol, as mentioned in Table 2, and their 2D interactions with the target protein active site residues are shown in Figure 1.

**TABLE 2:** Docking score of five compounds of *Ganoderma lucidum* extract onto the target protein MARK4

Name of the compound	Pubchem ID	Docking score (kcal/mol)
Ganoderic acid A	471002	−9.1
Ganoderic acid D	14109405	−9.1
Ganoderic acid F	23247895	−8.3
Ganoderenic acid B	78074039	−8.7
Ganoderenic acid D	76378890	−10.3

All the five ligands showed substantial interactions with the defined active site residues of the protein. The predominant interactions were hydrophobic, whereas the number of hydrogen bonds ranged from 2–6, with the least number of hydrogen bonds formed between MARK4 and 471002, and maximum hydrogen bonds between MARK4 and 76378890. Based on the promising docking interaction results, all the five screened molecule‐target protein complexes were subjected to the MD simulations.

### 3.3 Molecular dynamics simulation

**FIGURE 2:**
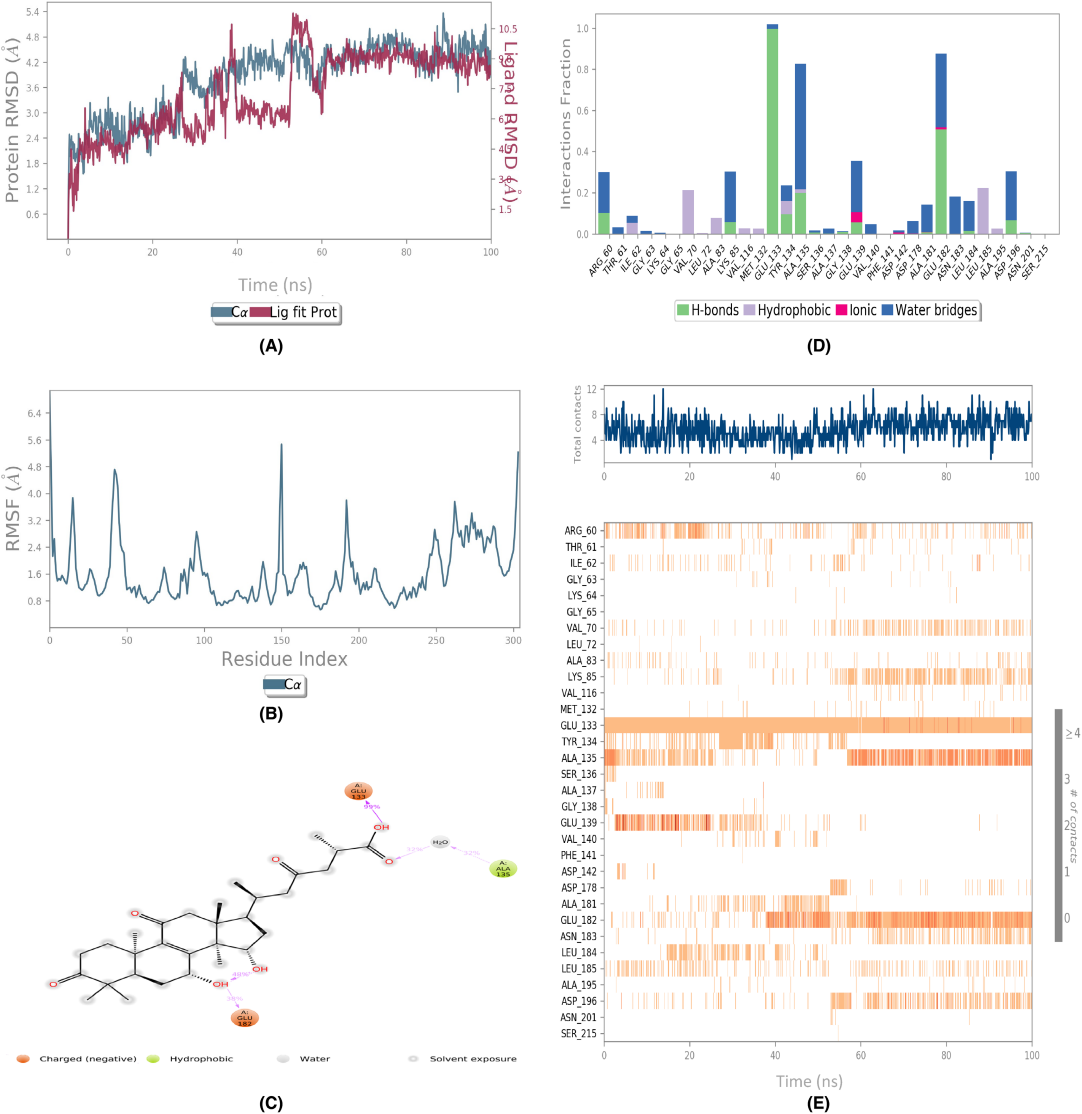
The MDS trajectory analysis of the MARK4 (5ES1)‐471002 complex. (A) RMSD, (B) RMSF, (C) protein‐ligand contact summary, (D) protein‐ligand contact histogram, (E) protein‐ligand contact timeline. MDS, Molecular Dynamics simulations; RMSD, root mean square deviation; RMSF, root mean square fluctuation.

**FIGURE 3:**
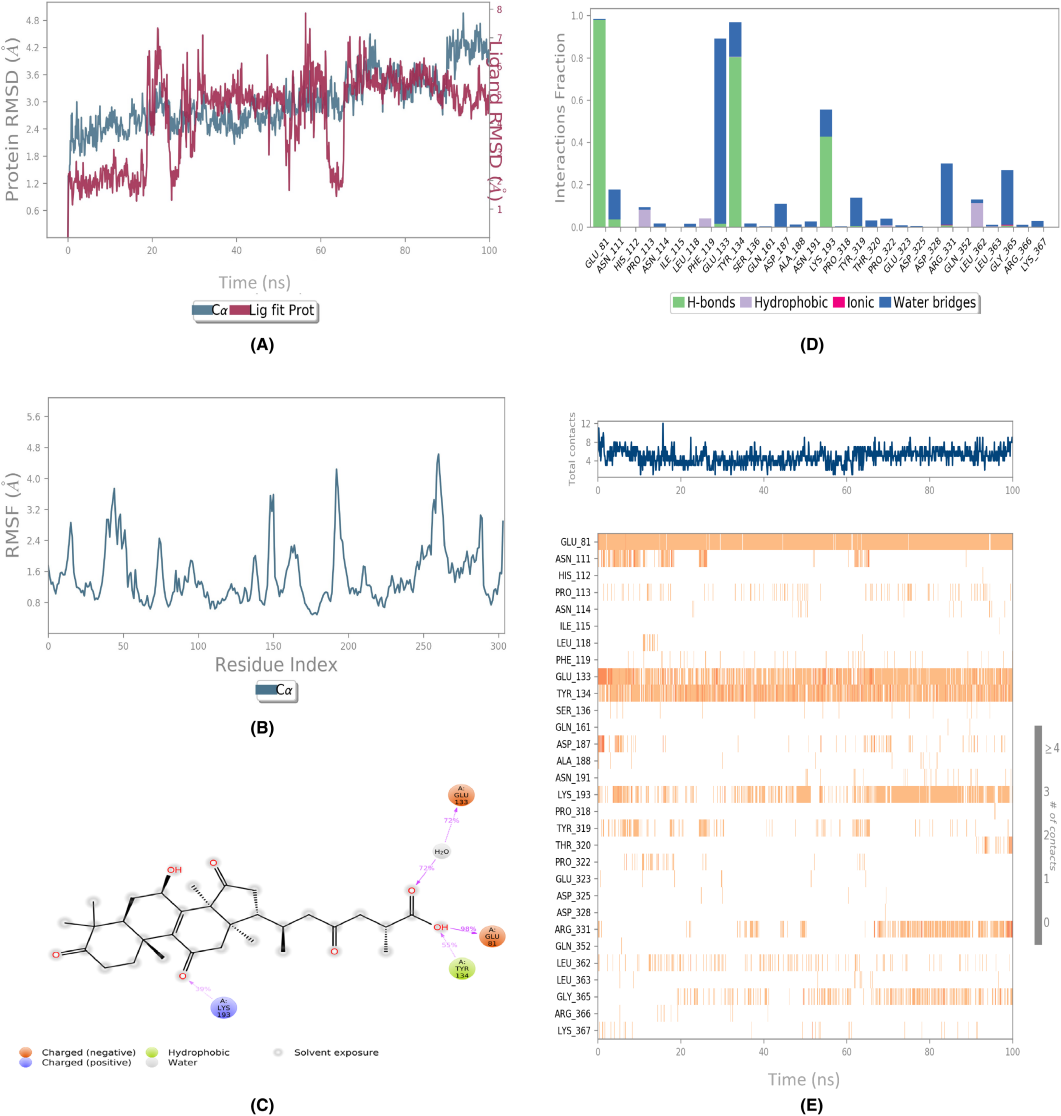
The MDS trajectory analysis of the MARK4 (5ES1) ‐ 14109405 complex. (A) RMSD, (B) RMSF, (C) protein‐ligand contact summary, (D) protein‐ligand contact histogram, and (E) protein‐ligand contact timeline. MDS, Molecular Dynamics simulations; RMSD, root mean square deviation; RMSF, root mean square fluctuation.

**FIGURE 4:**
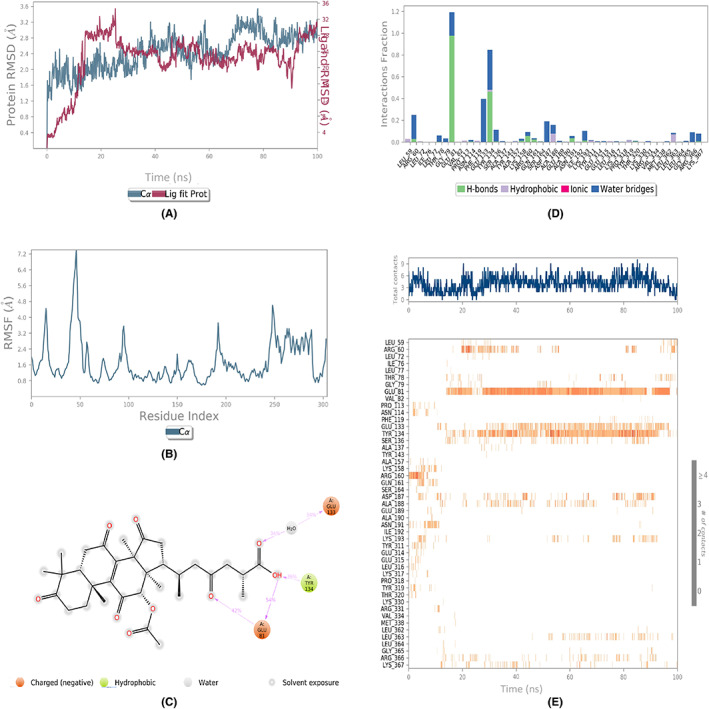
The MDS trajectory analysis of the MARK4 (5ES1) ‐ 23247895 complex. (A) RMSD, (B) RMSF, (C) protein‐ligand contact summary, (D) protein‐ligand contact histogram, and (E) protein‐ligand contact timeline. MD, Molecular Dynamics simulations; RMSD, root mean square deviation; RMSF, root mean square fluctuation.

**FIGURE 5:**
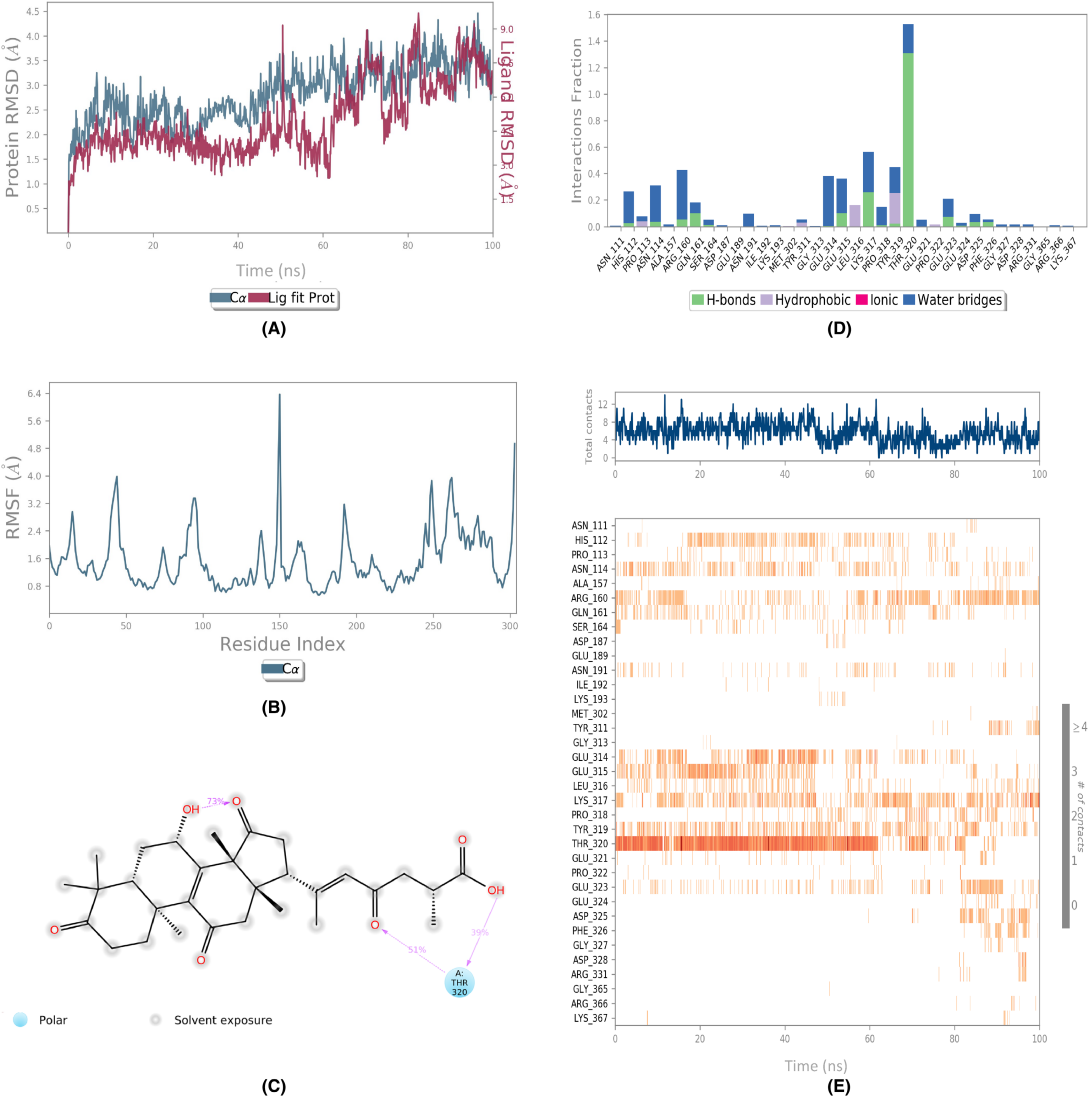
The MDS trajectory analysis of the MARK4 (5ES1)‐76378890 complex. (A) RMSD, (B) RMSF, (C) protein‐ligand contact summary, (D) protein‐ligand contact histogram, and (E) protein‐ligand contact timeline. MD, Molecular Dynamics simulations; RMSD, root mean square deviation; RMSF, root mean square fluctuation.

**FIGURE 6:**
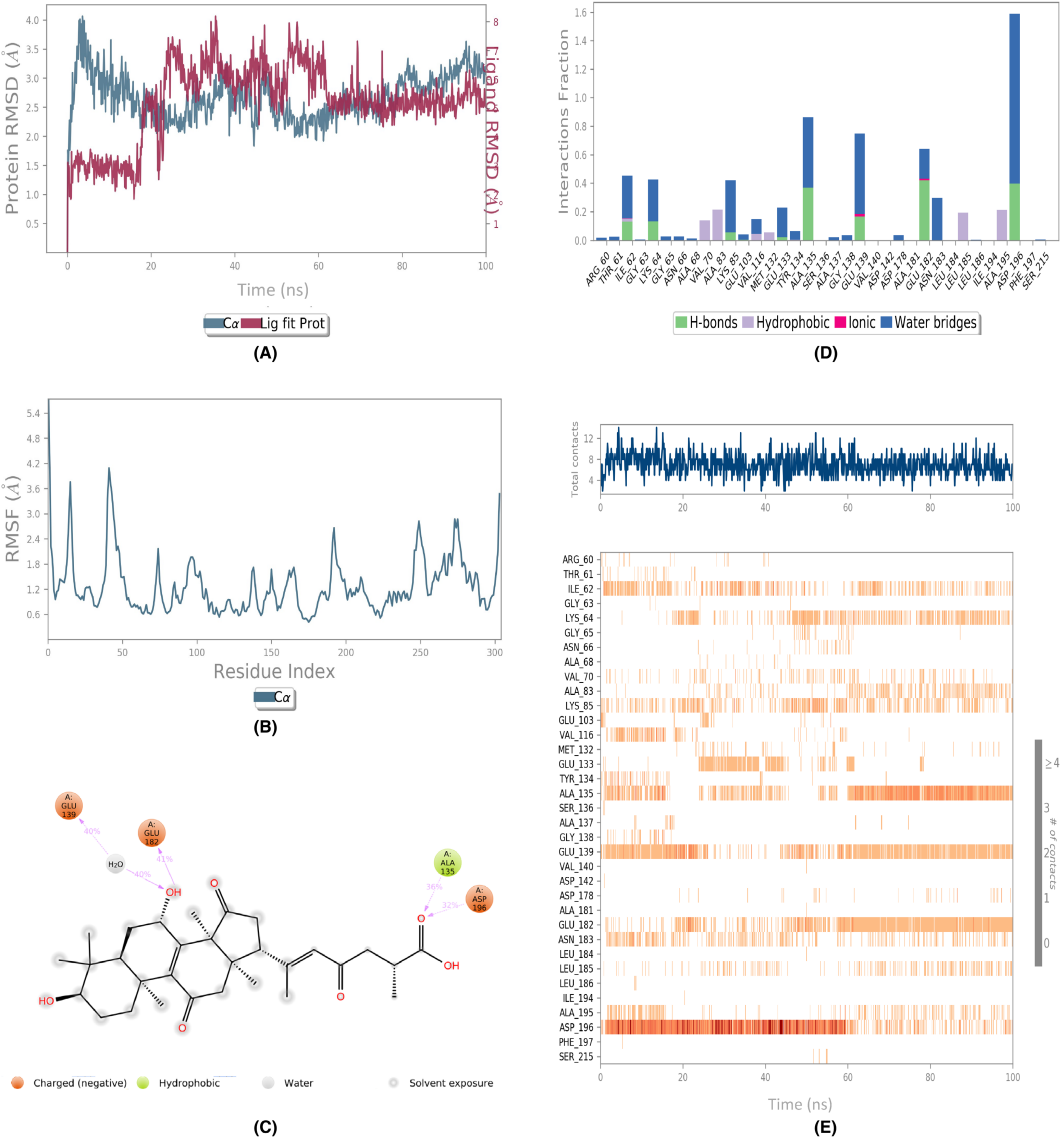
The MDS trajectory analysis of the MARK4 (5ES1)‐78074039 complex. (A) RMSD, (B) RMSF, (C) protein‐ligand contact summary, (D) protein‐ligand contact histogram, and (E) protein‐ligand contact timeline. MD, Molecular Dynamics simulations; RMSD, root mean square deviation; RMSF, root mean square fluctuation.

The assessment of the dynamic behavior of the protein as a result of the ligand binding was done by running the MD simulations. The analyses of the trajectories included root mean square deviation (RMSD), root mean square fluctuation (RMSF), and protein‐ligand contact mapping. The RMSD of all the complexes (Figures 2, 3, 4, 5, 6) equilibrated within the first 20 ns and ranged from 1.5 to 5 Å, with some internal fluctuations observed during the last 20–30 ns, as in the case of 1410905 and 78074039 in complex with MAPK4. The overall change in RMSDs to the initial frame was well within 5 Å and denoted the stable interactions. The maximum fluctuations in the RMSF values (Figures 2, 3, 4, 5, 6) were observed in the N‐ and C‐terminal of MAPK4 in complex with 471002 and 78074039, whereas high volatility was observed in the C terminal of MAPK4 in complex with 76378890. Additionally, higher fluctuations in the loop regions were observed in all the cases, whereas the RMSF values for the active site residues ranged from 1.6–2.4 Å for all the MAPK4‐ligand complexes asserting the regular interactions formed between the protein‐ligand complexes. The protein‐ligand interactions are critical to assessing the stability of the complex. The predominant interactions (Figures 2, 3, 4, 5, 6) were found to be H‐bonds followed by water bridges in all the complexes, whereas few ionic interactions were observed in the cases of 471002 and 78074039. As evident from Figures 2, 3, 4, 5, 6, the maximum interaction of the active site residues of MAPK4 was regarded in the instances of 471002 and 78074039; whereas the other ligands formed negligible interactions with the active site residues and instead interacted with the accessory residues near the active site.

**FIGURE 7:**
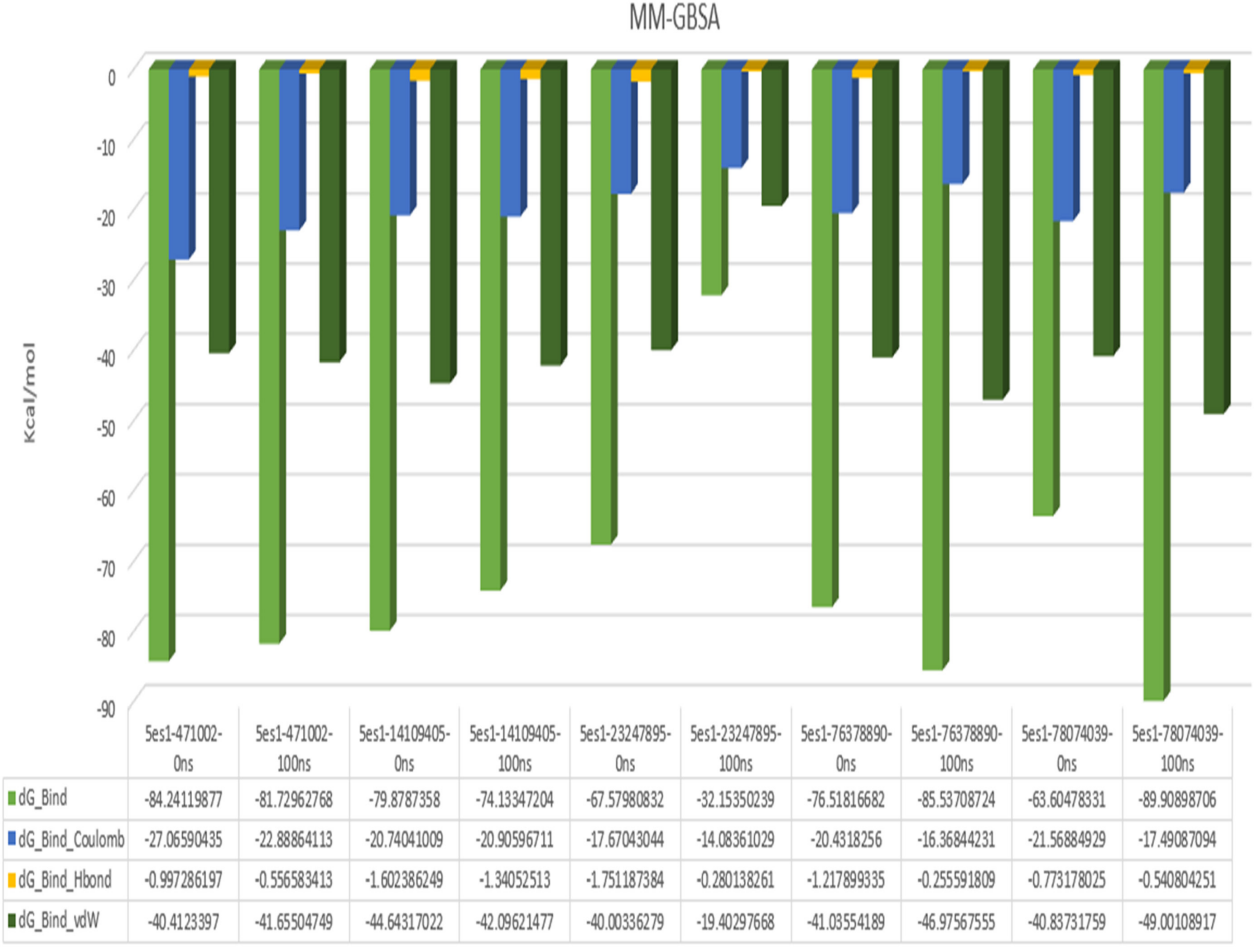
MMGBSA binding free energy calculation for the initial and final pose for the selected ligand‐MARK4 complexes. MMGBSA, molecular mechanics generalized born surface area.

The further validation of the protein‐ligand binding affinity was done by calculating the MMGBSA binding free energies for the complexes (Figure 7). The best two identified molecules, 78074039 and 471002, showed the highest binding affinity and interactions with critical active site residues after 100 ns with the energy scores of −89.80 and −81.72 kcal/mol, respectively, whereas the binding affinity of other ligand molecules were −85.53, −74.13 and −32.15 kcal/mol for 76378890, 1410905, and 23247895, respectively.

## 4 DISCUSSION

In structure‐based drug design, molecular docking is the most extensively utilized technique because it allows small compounds to interact with targets at the molecular level. Using this technique, we can better understand small molecule behavior in specific protein targets and predict the structure of ligand‐receptor complexes, which is critical for drug development. In this study, five compounds of *Ganoderma lucidum* extract were selected, which include ganoderic acid A, ganoderic acid D, ganoderic acid F, ganoderenic acid D, and ganoderenic acid B. These compounds showed the binding affinity of −9.1, −9.1, −8.3, −8.7, and −10.3 kcal/mol, respectively, with MARK4. Further validation was done by MD simulations and MMGBSA free energy calculations. It was predicted that ganoderic acid A and ganoderenic acid B might act as the two promising Reishi mushroom's compounds against MARK4. *Ganoderma lucidum* has medicinal properties and is even used for cancer but this study focusses on AD. Based on previous studies, ganoderic acid A is a kind of lanostane type triterpenoid, which alleviate neuroinflammation in AD mice via regulating the imbalance of the Th17/Tregs axis.^29^ Ganoderic acid A reduces Aβ42 levels in microglia cells by enhancing autophagy via the Axl/Pak1 signaling pathway. Ganoderic acid A ameliorates cognitive deficiency in the AD mouse model and even reduces Aβ42 in mouse.^30^ Yue et al (2021) showed that ganoderic acid A attenuates LPS‐induced neuroinflammation in BV2 microglia by activating the farnesoid X receptor (FXR).^31^ Nanhui et al (2020) showed that *Ganoderma lucidum* triterpenoids improve cognitive improvement by alleviating neuronal damage and inhibiting apoptosis in the hippocampus tissues and cells in AD by inhibiting the ROCK signaling pathway.^20^ Therefore, these previous results support this study, and no research study is available for ganoderenic acid B. Thus, making ganoderenic acid B a novel compound which can be focused upon and can be further studied for preclinical studies. This computational  study has shed a new light on the roles of ganoderenic acid B and ganoderic acid A as novel inhibitors of the MARK4 protein.

## 5 CONCLUSION

*Ganoderma lucidum* is known to exhibit cytotoxic, hepatoprotective, antioxidative, anticancer, and antinociceptive activities. This study predicted that *Ganoderma lucidum* could even be used to treat neurological disorders, like AD. The potential use of the best‐identified molecules ganoderic acid A and ganoderenic acid B against AD can be studied further using in vitro and in vivo models, on the basis of this study's computational validations.

## AUTHOR CONTRIBUTIONS

*Manuscript writing*: Ahmad. *Idea generation*: Ahmad. *Formal analysis*: Singh. *Methodology*: Singh. *Result*: Singh. *Review and editing*: Soni and Tandon.

## ACKNOWLEDGEMENTS

None.

## FUNDING INFORMATION

No funding was receieved for this study.

## CONFLICT OF INTEREST

The authors declare no conflict of interest.

## Data Availability

The data generated during this study are included in this article.
